# Supplementary material for: Coherent surface-to-bulk vibrational coupling in the 2D topologically trivial insulator Bi2Se3 monitored by ultrafast transient absorption spectroscopy
Source: Sci Rep. 2022 Mar 18;12:4722. doi: 10.1038/s41598-022-08513-5 (PMC8933573; doi:10.1038/s41598-022-08513-5)
Supplement: Supplementary file 1 — Supplementary Information. [file 41598_2022_8513_MOESM1_ESM.pdf]

Supporting Information

Coherent surface-to-bulk vibrational coupling in the 2D topologically trivial insulator Bi_2Se_3 monitored by ultrafast transient absorption spectroscopy

Yuri D. Glinka [1,2,*], Tingchao He [3,*], Xiao Wei Sun[1.4,*]

[1] Guangdong University Key Lab for Advanced Quantum Dot Displays and Lighting, Shenzhen Key Laboratory for Advanced Quantum Dot Displays and Lighting, Department of Electrical and Electronic Engineering, Southern University of Science and Technology, Shenzhen 518055, China
[2] Institute of Physics, National Academy of Sciences of Ukraine, Kyiv 03028, Ukraine
[3] College of Physics and Energy, Shenzhen University, Shenzhen 518060, China
[4] Shenzhen Planck Innovation Technologies Pte Ltd., Longgang, Shenzhen 518112, China

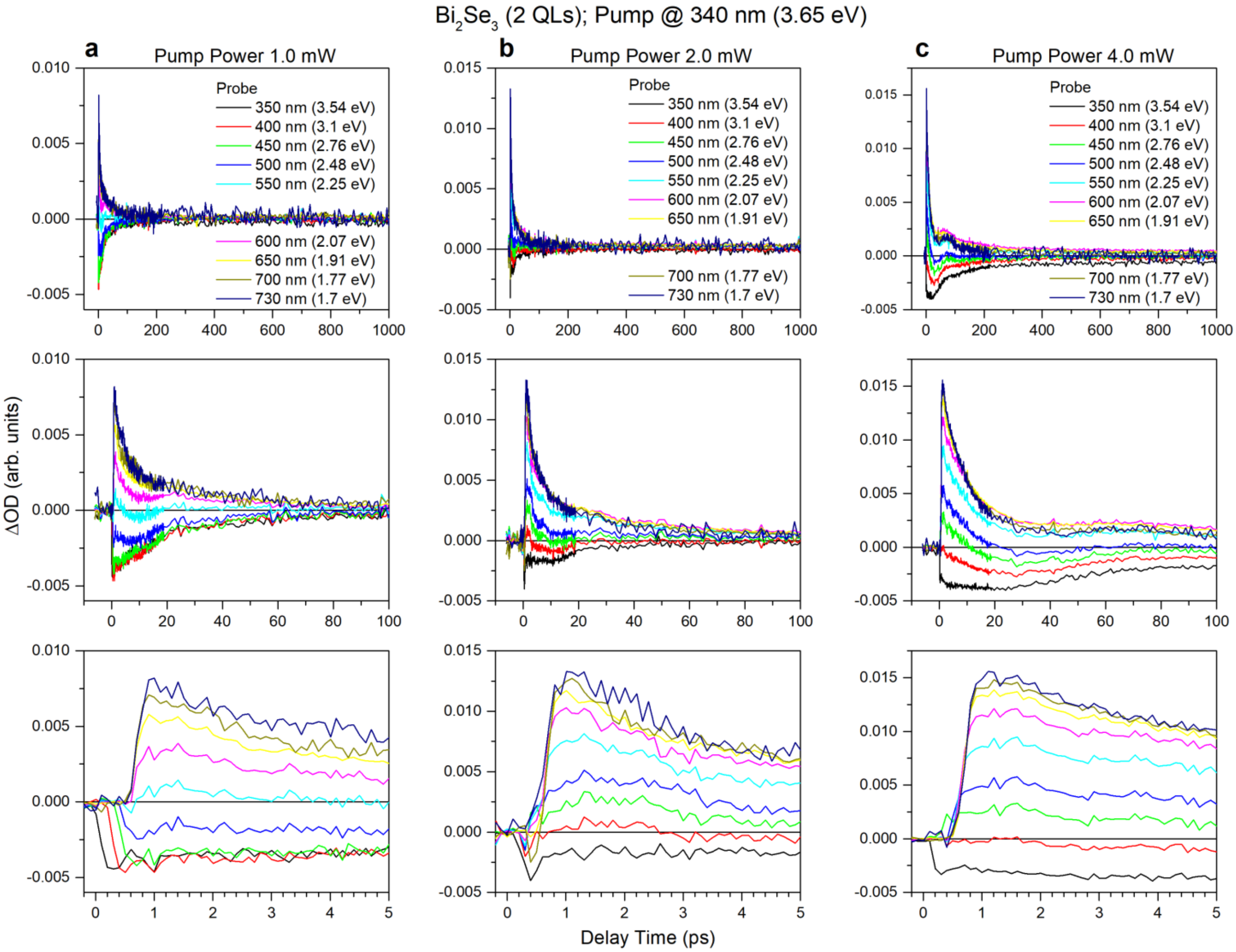

Fig. 1S. The pump–probe traces of the topologically trivial insulator phase of the 2D TI Bi_2Se_3 (2 QL film thickness): The pumping power effect (an extension of Fig. 4 in the main text for higher pumping powers). (a), (b), (c) The pump–probe traces measured with the 340 nm (3.65 eV) pumping of different powers, as indicate for each of the columns. The pump-probe traces in columns present the same data sets but plotted with different timescales. The traces were measured at the specific probing wavelengths, as indicated by the corresponding colors.

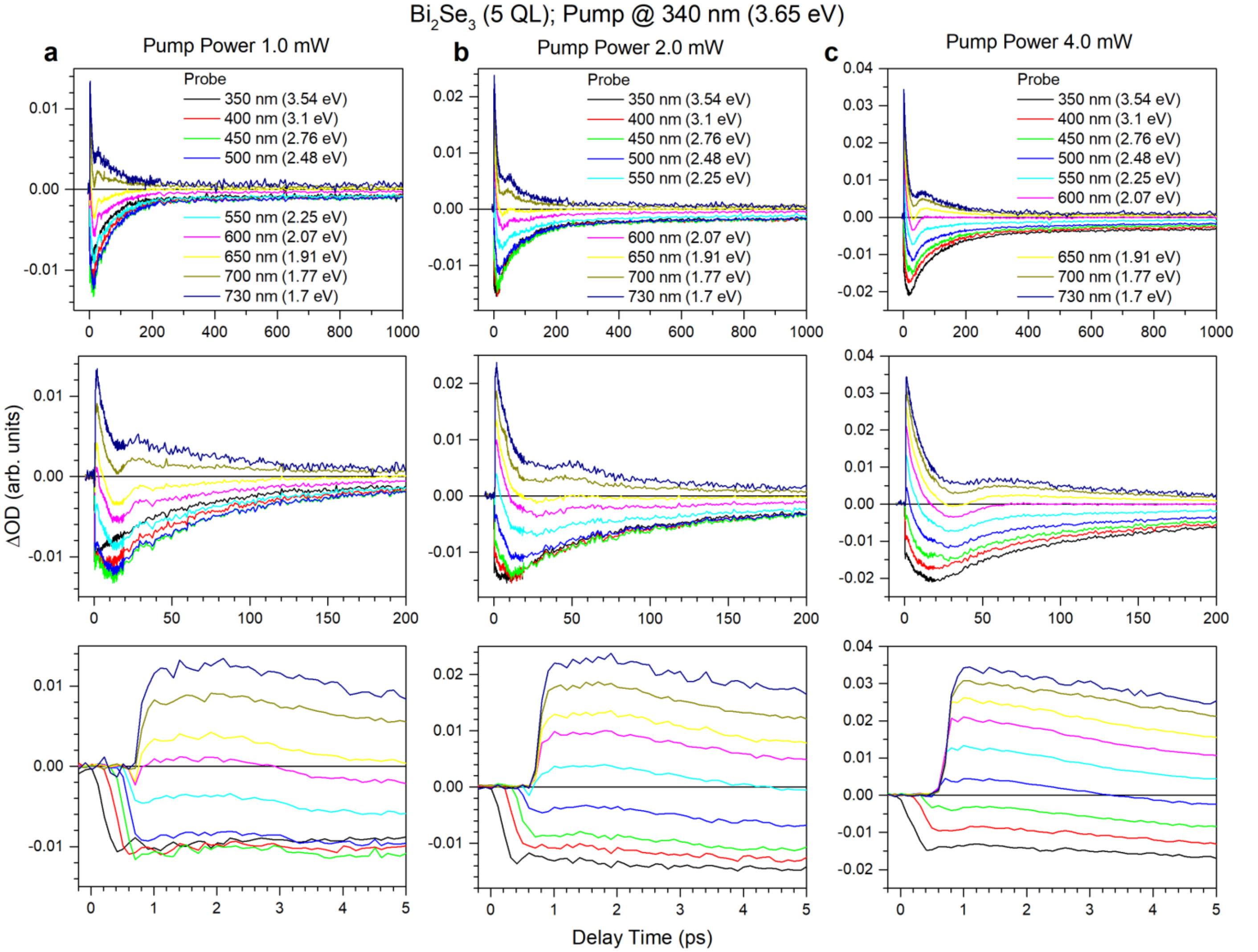

Fig. 2S. The pump–probe traces of the gapped topologically nontrivial insulator phase of the 2D TI Bi_2Se_3 (5 QL film thickness): The pumping power effect (an extension of Fig. 5 in the main text for higher pumping powers). (a), (b), (c) The transient pump–probe traces measured with the 340 nm (3.65 eV) pumping of different powers, as indicate for each of the columns. The pump-probe traces in columns present the same data sets but plotted with different timescales. The traces were measured at the specific probing wavelengths, as indicated by the corresponding colors.
